# Supplementary material for: Genetic Risk and Phenotype Correlation of Primary Open-Angle Glaucoma Based on Rho-Kinase Gene Polymorphisms
Source: J Clin Med. 2021 May 1;10(9):1953. doi: 10.3390/jcm10091953 (PMC8124732; doi:10.3390/jcm10091953)
Supplement: Supplementary file 1 [file jcm-10-01953-s001.zip › jcm-1191512-supplementary.pdf]

Table S1. List of Genotyping Primers

rs#	Allele Specific Primer-1	Allele Specific Primer-2	Locus Specific Primer	Specific Target Amplification Primer
rs1006881	AGGTTAATGAACGATTCTTACAGGAAGT	GGTTAATGAACGATTCTTACAGGAAGC	GCCTGGCCTGTAGTGATGC	TCATGAATGAATGGAACCATTAGATGAA
rs10083915	AGAATTGTTTAGCAATTTGAAAAAGTACAAT	AGAATTGTTTAGCAATTTGAAAAAGTACAAC	AATTATAAGTGAATATATTTTGATGTATTAAAGAAGGTGA	AGAATTGTTTAGCAATTTGAAAAAGTACA
rs10178332	CTTCTTCTGTTACTCCCACAGTATA	CTTCTTCTGTTACTCCCACAGTATC	GGGTATGAGTAGGACACATTCAAATAGAACA	CGGGAGTTTACACCCATTCAAATA
rs10929732	TCTGAATTGTGGAGTGGAATAAATCTGT	CTGAATTGTGGAGTGGAATAAATCTGC	ACGGTGCTATTAATCTGGATTTCA	CGCCAGAGCCAAAAAATCAG
rs1130157	TTTTATGGTAGATGAGAAAATTGTGAAAAAAATCAA	TTTTATGGTAGATGAGAAAATTGTGAAAAAAATCAG	ACAACATCATAGTCTTCTGCCTTCATCTG	TGGTGATCTTTTTATGGTAGATGAGAAAAT
rs11873284	GGCAATCAAAAATGTCTTCTCGACT	GGCAATCAAAAATGTCTTCTCGACC	GTATTTCTATTGTACTACTTAGAGCAGTCATTCTTAATTG	CACACGCCTTTCTCCAGTTAT
rs1481280	GGATATCATTTCATGAATAACTAGATGGCAGA	GATATCATTTCATGAATAACTAGATGGCAGC	TCCCAATCTCTCTGTTAAACCCAAACT	ACACTACATCTGAGGTCCTTTGG
rs1515210	TGGAAGCCTTAGCAAGACACC	TGGAAGCCTTAGCAAGACACG	GAGTGGAATTTATTTTCCATCTTTTGGAGTACA	GGCCACATGAGAAAAGAGTGAT
rs190769228	GAAGCAGACAAAAAACGAAATTTGGAAC	GAAGCAGACAAAAAACGAAATTTGGAAA	TGATGAGTAATTAATATTCTACACCACAAACCATCA	GCAGAATATCAGAGGAAAGCTGATC
rs202027620	ACAGGAACTTACGGAAAAAGATGCTAT	CAGGAACTTACGGAAAAAGATGCTAC	GCACCAAGAATTTGACTTACAGAAGCA	CAAAGAGATGATGGCTAGACACAAA
rs2127958	TGTTTCTCCAAAAATATGTCTGTTAGGTTTAG	GTGTTTCTCCAAAAATATGTCTGTTAGGTTTAA	TCATTTTTAGGTAAATTTTCAAAACCTAAATGGTTATTAT	CCATAGTTTTCAATGGATTTTGAGGG
rs2230773	CCAGGGGCTATTGGCAAAG	CCAGGGGCTATTGGCAAAA	CAGATTCTGCCTTTTTTTGGGAAGAAAGA	GTATGCTTACCTGAACCACCC
rs2271621	GGTCGGACATGAAATAACTTGCTATAAAAAAG	GGTCGGACATGAAATAACTTGCTATAAAAAAT	TAATTTGATCCTTGGGTGATATTTTATTCCTTTATTCAAA	ACACATCTGTCTGTGTAACTGGT
rs2847081	GGGGTTTCAGCTTAAATTAACCCG	GGGGTTTCAGCTTAAATTAACCCA	AGTAACTATGAGTTTGTCATTTAAGTTCTTTTACTTATTT	GGTTCACTCATGACCTGTCATT
rs288979	ATTCCCTGTGAATGGCCCATAT	TCCCTGTGAATGGCCCATAC	CAACAAAAAATTACAAAGCATGTAAAGAAGCAAGA	CGCCTGGTGGTTTCTAACATTT
rs288980	TGTTAATAAAAAAGGGCAGTAATATAAAAAATGGAC	TGTTAATAAAAAAGGGCAGTAATATAAAAAATGGAT	TCTGGAAGACTTGGTCCACATCAAA	GTGATTAAGACTAATGCAGTGAGGT
rs34945852	AGAAATTGACCCAGCAGATGATCAT	AGAAATTGACCCAGCAGATGATCAA	ACTGCCTGCATTTCATTCAGTTCTTTC	TGGAGCTTAAATCTGAACGTGAGA
rs35768389	AGCAGCTGGAATCTAACAATAGAGT	CAGCAGCTGGAATCTAACAATAGAGA	GCAGTCTCCAGCAGGCAGTT	AGGCAGAAAGTTCAAAACAGATTCA
rs35996865	AGTATTTTATGGCTCCCCTGACC	CAGTATTTTATGGCTCCCCTGACA	TGCAGCGCTCAGCCCT	TCTATTGACTGTATCTTCCGGAGTC
rs3771106	CAAAGGAGAACATTTCTAACAACCTAAGTATTTT	AAAGGAGAACATTTCTAACAACCTAAGTATTTC	AGAGTTGTATATTGAATTTAGTGTTTAAAAGTATTACATA	AGCAAAACATTTTTAATGAAAGAAACAAAGG
rs6755196	ACTCATGTGGCTAATCACACACAA	CTCATGTGGCTAATCACACACAG	TGAGTTTGGGCTGAATGAAGACTACT	TCCATAGCTTCAACTTACTCATGT
rs75122528	CGCGCCTGGTTGCATT	CCGCGCCTGGTTGCATA	CTTGGTGGAGGTCGTATACATCTTTCA	CAGGCATGAGCCACCG
rs8089184	GAGGTTAAATTCATTACTTTTATAGAAAGTTTGCG	AGAGGTTAAATTCATTACTTTTATAGAAAGTTTGCA	ACTTAATCATATTCTTAAAAACACAATTAACTTTAAATGA	TCAACATGTGAGAGGTTAAATTCATTACT
rs965665	CCTATCACAGCTCCTTCTCCAG	CCTATCACAGCTCCTTCTCCAC	GCAGGTTATTTCTGTTAGTCACGGGT	CCTAGACTTATCCTACCTCCCAT
